# Compact high-gain circularly polarized patch antenna based on TM30/TM03 mode

**DOI:** 10.1371/journal.pone.0321091

**Published:** 2025-05-06

**Authors:** Hoang Nguyen-Huy, Duc-Nguyen Tran-Viet, Phuong Kim-Thi, Hung Tran-Huy

**Affiliations:** 1 Faculty of Electrical and Electronic Engineering, PHENIKAA University, Yen Nghia, Ha Dong, Hanoi 12116, Vietnam; 2 Faculty of Radio-Electronic Engineering, Le Quy Don Technical University, Hanoi 11917, Vietnam; 3 Faculty of Electrical and Electronic Engineering, Thuyloi University, Hanoi, Vietnam; Parul University, INDIA

## Abstract

This paper presents a circularly polarized (CP) microstrip patch antenna with high gain and compact size characteristics. For high gain radiation, the principle is to employ high-order mode patch. By using diagonal feed and unequal center crossed slot, two orthogonal modes including TM_03_ and TM_30_ modes are generated with equal magnitude and $90^{\circ }$ phase difference. Thus, the high-order mode patch antenna can radiate CP waves with high gain. To achieve small lateral dimensions with low back radiation, the patch is surrounded by a substrate integrated waveguide (SIW) cavity. For validation, measurement is implemented on an antenna prototype. The final design has overall dimensions of 1.27λ × 1.27λ × 0.03λ at the center operating frequency. The measured operating bandwidth, in which reflection coefficient and axial ratio are respectively less than –10 dB and 3 dB, is from 5.435 to 5.475 GHz. Within this band, the antenna exhibits high gain radiation of about 12 dBi. Besides, compared to the antenna without SIW cavity, the back radiation of the proposed design can be decreased by two times.

## Introduction

Microstrip patch antennas have been widely used in various types of communication systems due to their low profile, light weight, and ease of integration. However, low gain radiation makes them less attractive to the modern systems, in which long-distance communication is required. Besides, using circularly polarized (CP) antenna has more benefits than using linearly polarized (LP) antenna since it can reduce polarization mismatch and multipath interference as well.

Traditional microstrip patch antennas with CP radiation suffer from a critical drawback of low gain radiation [1–3], which is less than 8 dBi. Various approaches have been reported in the open literature to improve the gain of patch antenna. A typical scheme is increasing the number of radiating elements [4, 5]. However, the structures’ dimensions and complexity will also increase. Another approach is introduced by loading special structures, such as frequency selective surface (FSS) or dielectric superstrate [6–8]. Nonetheless, they will undoubtedly increase the antenna’s profile to at least quarter-wavelength at the operating frequency. Similar drawbacks can be observed by the techniques of using stacked radiating elements as reported in [9–11]. One of the potential solutions for gain enhancement while retaining the low-profile structure is a combination between the metasurface (MS) and microstrip patch radiator [12–15]. Overall, such above-mentioned methods have similar problems of high profile and complicated structures.

Currently, using high-order mode is an effective solution for gain enhancement due to the increase of electrical size. This method does not require any additional structure. In [16, 17], multiple shorting pins are inserted within the patch to achieve a high gain of about 10.8 dBi. Alternatively, the use of high-order mode of TM_30_ patch antenna is also the way to realize high gain property [18–20]. Although a high gain of about 12.0 dBi can be achieved, they suffer from disadvantages of large size and/or complicated structure.

In this paper, a single-feed CP patch antenna with high gain radiation, compact size, and simple structure based on the high-order TM_30_/TM_03_ mode is proposed. Four open slots are inserted into the square-shape patch to not only reduce the grating lobe but also reduce the antenna dimensions. Next, a substrate integrated waveguide (SIW) cavity is used to improve the front-to-back ratio by reducing the back radiation. In comparison with the related works, the proposed design possesses several advantages of compact size, simple structure, as well as high gain property.

## High-order mode CP patch antenna

**Fig 1:**
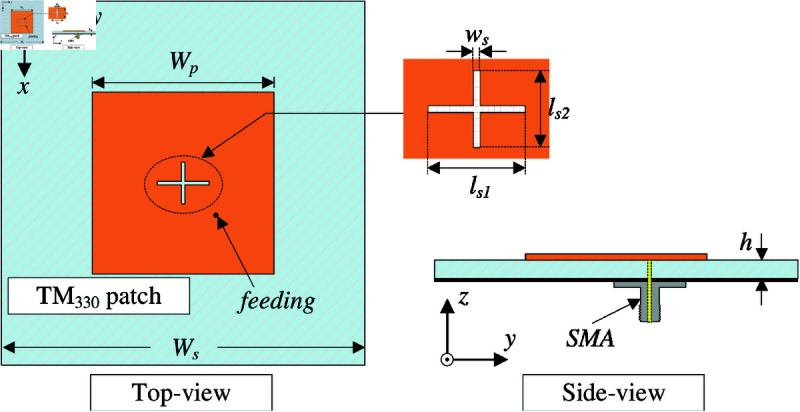
Geometry of the high-order mode CP patch antenna.

The geometrical configuration of the high-order mode CP patch antenna is presented in Fig 1. The antenna structure is simply comprised of a square patch and a ground plane. The patch and the ground are printed on top and bottom layers of a Taconic TLY-5 substrate with a dielectric constant of 2.2 and loss tangent of 0.0009. The patch is diagonally fed, and an unequal crossed slot is loaded at the patch’s center. The antenna is modelled and characterized using the commercial full-wave simulation software, High Frequency Structure Simulator (HFSS). The optimal design parameters are as follows: *W*_*s*_ = 100, *h* = 1.52, *W*_*p*_ = 51.4, *l*_*s*1_ = 14.6, *l*_*s*2_ = 12, *w*_*s*_ = 0.8 (unit: mm).

The radiating patch with overall dimensions of *W*_*p*_ × *W*_*p*_ is designed to operate at high-order mode of TM_03_. At the desired frequency (*f*_*r*_), the resonant length can be estimated based on the following equation [21]:

$$W_{p}=3\frac{\lambda_{eff}}{2}=3\frac{c}{2f_{r}\sqrt{\varepsilon_{eff}}}=3\frac{c}{2f_{r}}\frac{1}{\sqrt{\frac{\varepsilon_{r}+1}{2}+\frac{\varepsilon_{r}-1}{2\sqrt{1+h/W_{p}}}}}$$ (1)

where the light velocity is *c*, and $\varepsilon_{r}$ and $\varepsilon_{eff}$ are dielectric constant and effective dielectric constant, respectively. Thickness of the substrate is *h*, and $\lambda_{eff}$ is the wavelength in substrate. For the high-order mode patch, the patch’s size is much greater than the substrate’s thickness; therefore, $\varepsilon_{eff}$ can be approximately equal to $\varepsilon_{r}$.

**Fig 2:**
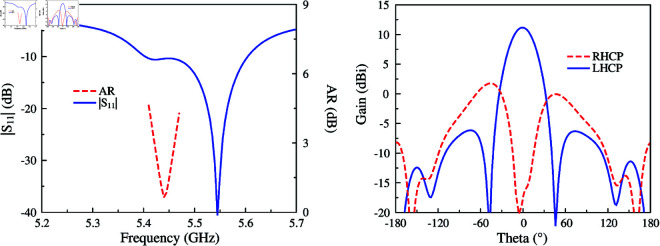
Simulated |*S*_11_|, AR and gain radiation patterns of the antenna.

**Fig 3:**
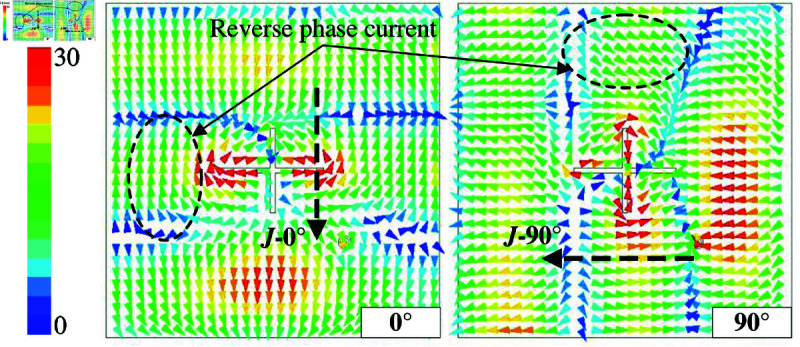
Simulated current distribution on the patch.

Fig 2 shows the simulated results in terms of reflection coefficient (|*S*_11_|), axial ratio (AR), and gain radiation pattern of the proposed antenna. It can be seen obviously that the antenna shows good operation at the desired frequency of 5.45 GHz, in which |*S*_11_| and AR are respectively less than –10 dB and 3 dB. For the gain radiation response, although the antenna radiates strong power in the broadside direction, the grating lobe is quite high. This is caused by the out-of-phase current flowing on the radiating patch. For a better demonstration, Fig 3 shows the simulated current distribution on the patch at different phases of $0^{\circ }$ and $90^{\circ }$. Overall, the main vector currents in both cases are orthogonal and when the phase changes, the vector current rotates in clockwise direction. This demonstrates the left-hand CP radiation. However, the middle current (dot-black circle) is out-of-phase with the other currents, leading to a high grating lobe level.

**Fig 4:**
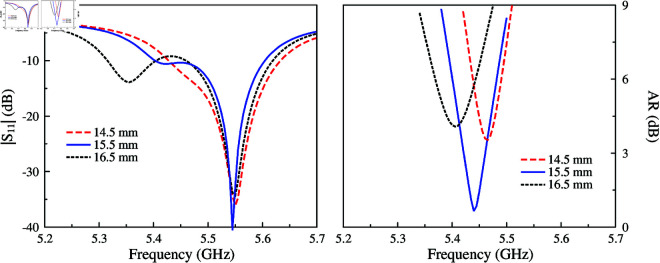
Simulated antenna performance for different values of *l*_*s*1_.

**Fig 5:**
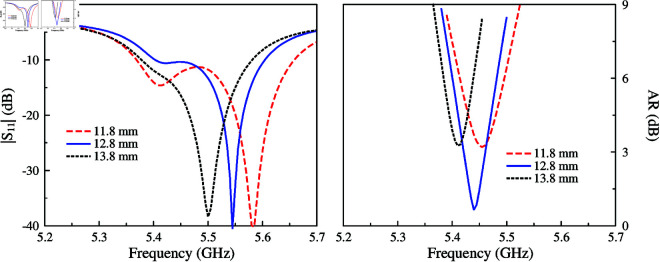
Simulated antenna performance for different values of *l*_*s*2_.

The AR optimization of this antenna strongly depends on the dimensions of the unequal crossed slot locating at the center of the patch. This crossed slot contributes to producing two orthogonal modes, TM_30_ and TM_03_. By tuning the slot’s length, the magnitudes and phases of these modes can be controlled so that they are equal in magnitude and $90^{\circ }$ phase difference. Figs 4 and 5 show the simulated |*S*_11_| and AR for different values of *l*_*s*1_ and *l*_*s*2_. In the |*S*_11_| profile, there are two resonances corresponding to the TM_30_ and TM_03_ modes. Tuning *l*_*s*1_ and *l*_*s*2_ will have a significant effect on the corresponding resonance, which shifts upwards or downwards depending on the length variation of the slot’s length. When the phase requirement is satisfied, the AR value under 3 dB can be obtained.

## High-order mode CP patch antenna with four open slots

**Fig 6:**
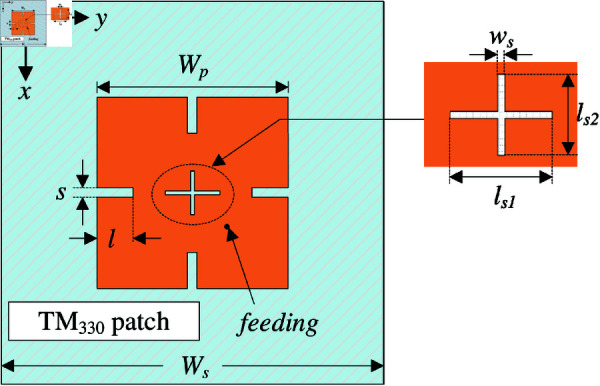
Top-view of the high-order mode CP patch antenna with four open slots.

Despite having high gain operation, the design in the previous section suffers from a critical drawback of high grating lobe, which significantly deteriorates the system’s performance. To overcome this drawback, the reverse phase current in the medial portion of the patch should be eliminated. In this context, the patch radiation is like two in-phase TM10/TM01 modes. To achieve this, four open slots are inserted along *x*– and *y*–direction, as shown in Fig 6. Noted that the width of such slots should be narrow so that the radiation from the circumventing current could be almost canceled with each other. Thus, the radiation of such slots will not have significant effect on the radiation of the patch. The antenna is optimized to achieve the best performance around 5.45 GHz. The optimal design parameters are as follows: *W*_*s*_ = 70, *h* = 1.52, *W*_*p*_ = 46, *l*_*s*1_ = 14.6, *l*_*s*2_ = 9.6, *w*_*s*_ = 0.8, *s* = 2.8, *l* = 9.6 (unit: mm).

**Fig 7:**
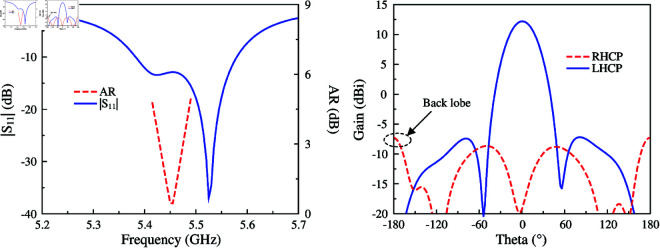
Simulated |*S*_11_|, AR and gain radiation patterns of the antenna with open slots.

**Fig 8:**
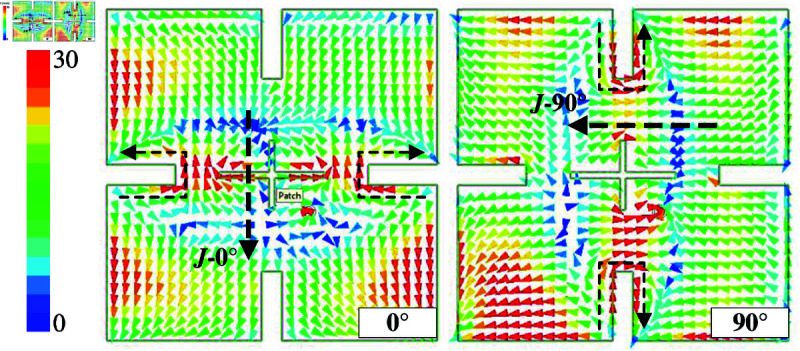
Simulated current distribution on the patch with open slots.

**Fig 9:**
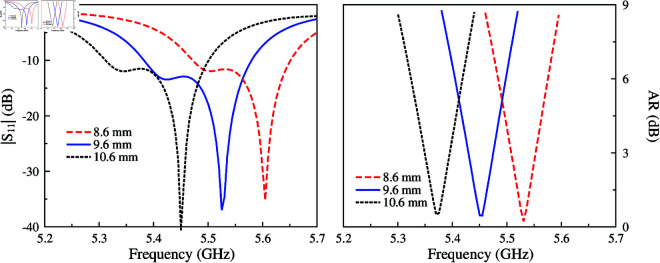
Simulated performance for different values of *l* of the antenna with open slots.

Further demonstration about the effect of four open slots is shown in Fig 7, which shows the simulated |*S*_11_|, AR, as well as gain radiation pattern of the high-order mode CP patch antenna with four open slots. The data indicates that the antenna well operates at 5.45 GHz with a broadside gain of 12.2 dBi. Meanwhile, the grating lobe is much lower than that of the design in the previous section, which is –7.4 dB compared to 3.4 dB. This is attributed to the elimination of the center reverse phase current on the radiating patch. Fig 8 shows the simulated current distribution on the patch at 5.45 GHz. As observed, the out-of-phase current is circumvented along the four open slots. Therefore, the grating lobe can be suppressed. It is also noted that due to the presence of these open slots, the electrical length of the antenna can be increased by increasing the slot’s length, resulting in the patch size reduction. The effect of the slot’s length (*l*) on |*S*_11_| and AR is shown in Fig 9. As seen, the operating frequency can be tuned by changing *l*. Increasing *l* leads to a longer electrical length; therefore, the operating frequency shifts towards the lower frequency range.

## High-order mode CP patch antenna with four open slots and SIW cavity

**Fig 10:**
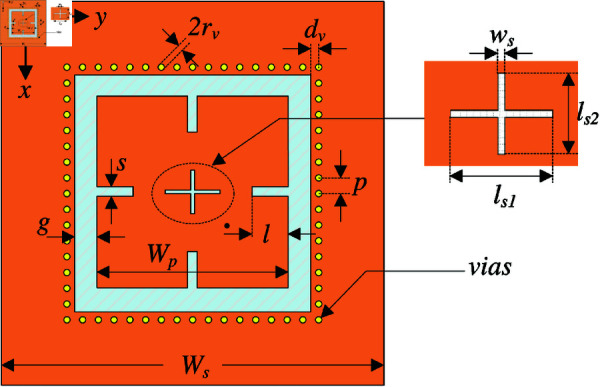
Top-view of the high-order mode CP patch antenna with open slots and SIW cavity.

Although compact size can be achieved when four open slots are employed, the back radiation of the antenna in the above-mentioned section is quite high of about –7.3 dB. This is due to the high diffracted power at the edges of the substrate. To reduce this diffraction, a SIW cavity is one of the effective solutions. Fig 10 shows the configuration of the proposed high-order mode CP patch antenna with four open slots and SIW cavity. The optimal dimensions of this design are as follows: *W*_*s*_ = 70, *h* = 1.52, *W*_*p*_ = 46, *l*_*s*1_ = 14.6, *l*_*s*2_ = 10.2, *w*_*s*_ = 0.8, *s* = 2.4, *l* = 9.6, *g* = 3.4, $r_{v}$ = 0.5, *p* = 1.4, $d_{v}=1$ (unit: mm).

**Fig 11:**
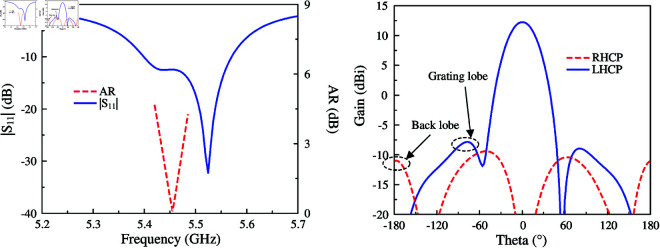
Simulated |*S*_11_|, AR and gain radiation patterns of the antenna with open slots and SIW cavity.

The simulated performance of the final antenna realization is illustrated in Fig 11 The antenna has good matching performance in the frequency range from 5.4 to 5.57 GHz. The AR values of less than 3 dB are from 5.43 to 5.48 GHz. Across this band, the broadside gain is about 12 dBi. In terms of gain radiation patterns at 5.45 GHz, the back lobe of the antenna with SIW cavity is about –11.2 dB, which is lower than that of the antenna without cavity. Meanwhile, the grating lobe is about –7.2 dB. The performance metrics of three different antennas are summarized in Table 1. The main lobe and operating BW shown in the comparison table are almost similar. In contrast, the grating lobe and back lobe of the final antenna are better than the others.

**Table 1:** Performance comparison among presented antennas.

Design	Operating BW (MHz)	Grating lobe (dB)	Back lobe (dB)	Main lobe (dB)
Patch antenna	55	3.4	–8.2	12.3
Patch antenna with four open slots	53	–7.4	–7.3	12.2
Patch antenna with four open slots and SIW cavity	50	–7.2	–11.2	12

## Measurement results

**Fig 12:**
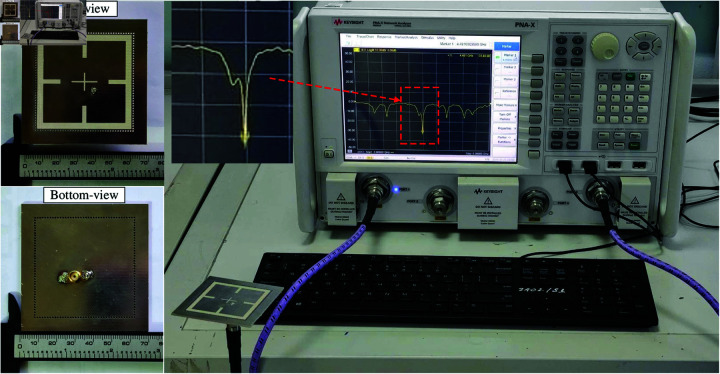
Photographs of the fabricated antenna prototype.

The proposed antenna is fabricated and measured to validate the design concept. The photographs of the antenna prototype are shown in Fig 12. The measurements are carried out using a Vector Network Analyzer and Anechoic Chamber. Overall, the simulation is fairly matched with the measurement.

**Fig 13:**
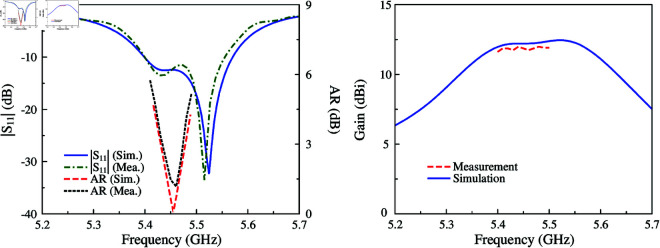
Simulated and measured |*S*_11_|, AR and gain of the proposed antenna.

**Fig 14:**
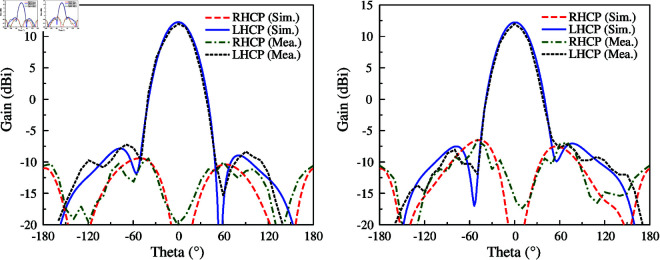
Simulated and measured gain radiation patterns of the proposed antenna in *x*–*z* and *y*–*z* planes.

The simulated and measured performance with respect to |*S*_11_|, AR, and broadside gain results are shown in Fig 13. Meanwhile, the simulated and measured gain radiation patterns in two principal planes are illustrated in Fig 14. As observed in the frequency range from 5.435 to 5.475 GHz, the antenna achieves good matching performance and low AR values of less than 3 dB. The measured broadside gain demonstrates that the fabricated antenna exhibits a high gain of around 12 dBi within the operating bandwidth. Meanwhile, the radiation patterns at 5.45 GHz are quite symmetrical around the broadside direction. The measured grating lobe is about –6.8 dB and the measured back lobe is approximately –10.5 dB.

Table 2 shows a comparison between the proposed antenna and several remarkable high gain antennas in the literature. According to the compared data, it is obvious that the proposed antenna has the lowest profile structure, small lateral dimensions, as well as simple structure with no additional feeding network and only single-layer. Several designs have wide bandwidth due to the use of complicated structure with large size and multiple layers to excite multiple modes. In terms of gain, the proposed design has comparable gain with the others. The use of FSS or dielectric substrate results in a very high structure profile [6, 7]. Meanwhile, lower profile and high gain can be achieved with the aid of MS layer; however, large lateral dimensions are critical drawback of the antennas in [12, 13, 15]. Compared to the similar method of using high order mode, the proposed antenna has lower grating lobe level and smaller size than the design in [18] while achieving similar front-to-back ratio (FBR). Besides, higher gain and/or less complexity are the advantages of the proposed design compared to the results in [16] and [19]. Noted that the design in [19] has wide bandwidth due to the use of chip coupler, which complicates the design and the insertion loss will affect the antenna gain.

**Table 2:** Performance comparison among high-gain CP antennas.

Ref.	Overall dimension	Antenna structure	No. of layers	BW (%)	Gain (dBic)	FBR (dB)
	1.50 × 1.50 × 0.05	2 × 2 patches + feeding network	2	17.6	11.5	16
	1.79 × 1.79 × 0.64	Patch + FSS layer	3	6.9	14.6	15
	1.48 × 1.48 × 0.64	Patch + dielectric superstrate	3	13.6	13.2	20
	1.22 × 1.22 × 0.07	Patch + MS layer	2	33.3	12.2	30
	1.87 × 1.87 × 0.05	Patch + MS layer	3	6.1	12.3	15
	1.42 × 1.42 × 0.08	2 × 2 patches + MS + feeding network	2	83.2	10.8	N/A
	1.27 × 1.27 × 0.03	Patch + shorting pins	1	~1.0	10.3	40
	1.53 × 1.53 × 0.03	TM_03_/TM_30_ Patch + SIW cavity	1	0.6	12.5	22
	0.79 × 0.79 × 0.03	TM_03_/TM_30_ Patch + Hybrid chip coupler	2	12.5	9.1	14.4
Prop.	1.27 × 1.27 × 0.03	TM_03_/TM_30_ Patch + SIW cavity	1	0.7	12	22.5

## Application of the proposed antenna for unmanned aerial vehicle

**Fig 15:**
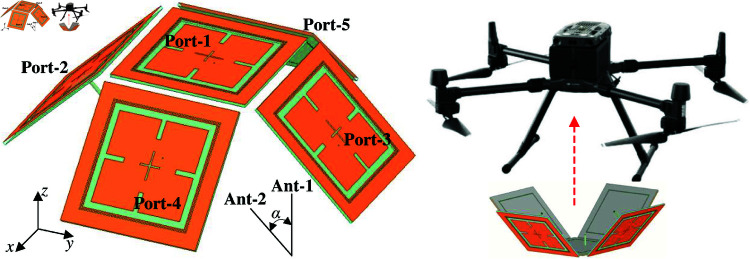
Geometry of the antenna array for UAV applications.

The proposed antenna possesses high gain radiation and CP radiation as well. It could be benefit for long-range communication systems. Besides, CP radiation also helps to increase the channel reliability by avoiding polarization mismatch. Nowadays, unmanned aerial vehicles (UAVs) have been widely used in various fields such as public safety, monitoring, security surveillance, and so on. In this context, air-to-ground communication is very important. By using the proposed antenna, an antenna array system consisting of five elements with titled angle of $\alpha =45^{\circ }$ is proposed in Fig 15. This antenna system is designed for covering the area downward the UAV-body. It can switch the radiation pattern in different directions, which allows wide air-to-ground communication coverage.

**Fig 16:**
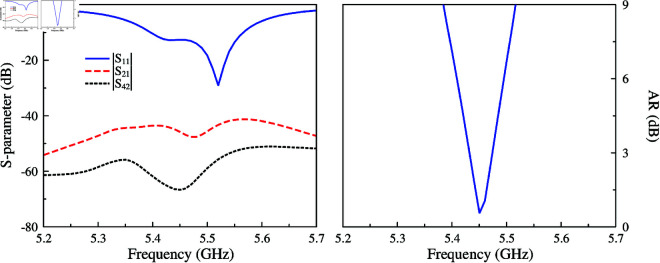
Simulated S-parameter and AR of the antenna array.

**Fig 17:**
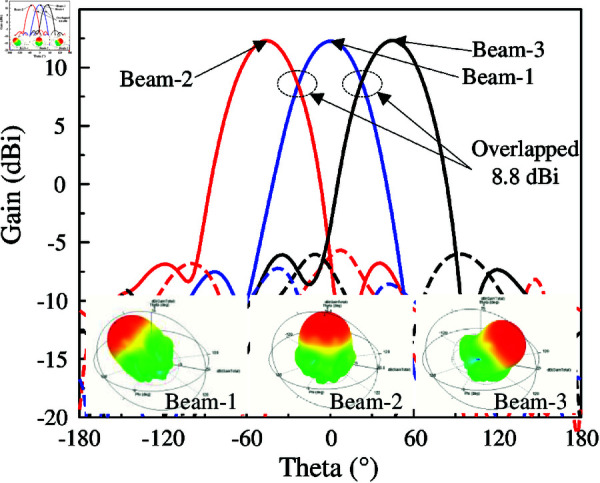
Simulated gain radiation patterns of the antenna array in *y*–*z* plane.

The simulated reflection and transmission coefficients, as well AR with Port-1 excitation of the antenna array are presented in Fig 16. It can be observed that the array system achieves a good reflection coefficient around 5.45 GHz. Meanwhile, the isolation is always better than 44 dB. The AR values are lower than 3 dB in the frequency range from 5.43 to 5.47 GHz, which demonstrates the CP radiation of the proposed design. The simulated gain radiation patterns are shown in Fig 17. Changing the excited port, the beam can be switched at $45^{\circ }$ in the *y*–*z* plane. The maximum gain is about 12 dBi and the gain at the overlapped patterns is approximately 9.0 dBi. Similar operation features can be achieved in the *y*–*z* plane due to the symmetrical array configuration.

## Conclusion

A high gain CP patch antenna with side-lobe reduction and size miniaturization is presented and investigated in this paper. The design evolution step is also given to better demonstration the working principle and optimization process of the proposed antenna. The design is first characterized using simulation and then validated by measurement. By using four-sided slots and four-crossed slots, the side-lobe level and lateral dimensions of the proposed antenna can be optimized. The measured data demonstrate that high gain of 12 dBi across the operating bandwidth from 5.435 to 5.475 GHz can be achieved with compact overall antenna’s dimensions of 1.27 λ × 1.27 λ × 0.03 λ.
